# U.S. Adolescent and Adult Women’s Experiences Accessing and Using Toilets in Schools, Workplaces, and Public Spaces: A Multi-Site Focus Group Study to Inform Future Research in Bladder Health

**DOI:** 10.3390/ijerph16183338

**Published:** 2019-09-10

**Authors:** Deepa R. Camenga, Sonya S. Brady, Cecilia T. Hardacker, Beverly R. Williams, Jeni Hebert-Beirne, Aimee S. James, Kathryn Burgio, Jesse Nodora, Jean F. Wyman, Amanda Berry, Lisa K. Low, The Prevention of Lower Urinary Tract Symptoms (PLUS) Research Consortium

**Affiliations:** 1 Department of Emergency Medicine, Yale School of Medicine, New Haven, CT 06519, USA; 2 Division of Epidemiology & Community Health, University of Minnesota School of Public Health, Minneapolis, MN 55454, USA; 3 Department of Education, Center for Education, Research and Advocacy, Howard Brown Health, Chicago, IL 60613, USA; 4 Department of Medicine, Division of Gerontology, Geriatrics and Palliative Care, University of Alabama at Birmingham, Birmingham, AL 35233, USA; 5 Division of Community Health Sciences, School of Public Health, University of Illinois at Chicago, Chicago, IL 60612, USA; 6 Division of Public Health Sciences, Department of Surgery, Washington University in St Louis School of Medicine, St Louis, MO 63110, USA; 7 Department of Medicine, University of Alabama at Birmingham and Department of Veterans Affairs, Birmingham, AL 35233, USA; 8 Department of Family Medicine and Public Health, University of California San Diego School of Medicine & Moores Cancer Center, La Jolla, CA 92093, USA; 9 School of Nursing, University of Minnesota, Minneapolis, MN 55455, USA; 10 Children’s Hospital of Philadelphia, Philadelphia, PA 19104, USA; 11 Department of Health Behavior and Biological Sciences, School of Nursing, University of Michigan, Ann Arbor, MI 48109, USA; 12 Minneapolis, MN 55414, USA

**Keywords:** bladder health, female, toilet, qualitative research, focus groups

## Abstract

The World Health Organization recognizes access to clean and safe toilets as crucial for public health. This study explored U.S. adolescent and adult cisgender women’s lived experiences accessing toilets in schools, workplaces, and public spaces. As part of the Prevention of Lower Urinary Tract Symptoms (PLUS) Research Consortium, we conducted 44 focus groups with female participants (n = 360; ages 11–93). Focus groups were stratified by age (11–14, 15–17, 18–25, 26–44, 45–64, 65+) and conducted across 7 geographically diverse U.S. sites from July 2017–April 2018. Using a transdisciplinary approach, we conducted conventional qualitative coding informed by our PLUS conceptual framework and used content analysis processes to identify salient themes. Across settings, toilet access was restricted by “gatekeepers” (i.e., individuals who control access to toilets). In contrast, self-restricting toilet use (deciding not to use the toilet despite biologic need to urinate) was based on internalized norms to prioritize school and job responsibilities over urination. In public spaces, self-restricting use was largely in response to lack of cleanliness. Across the life course, participants perceived gender disparities in the ability to easily access public toilets. Further research is needed to determine if and how these factors impact bladder health across the life course.

## 1. Introduction

The World Health Organization recognizes access to and use of clean and safe toilets as crucial for public health [1]. To date, public health efforts to improve toilet access and use have largely focused on the prevention of a variety of infectious diseases related to contaminated drinking water, with less public health attention on the need for toilet access to maintain bladder health and prevent lower urinary tract symptoms (LUTS) [1]. Toilet access, or the ability to approach or enter bathrooms for toileting, has been identified as a potential protective factor for bladder health [2,3]. Broadly defined, bladder health is “a complete state of physical, mental, and social well-being related to bladder function and not merely the absence of lower urinary tract symptoms (LUTS)” [4]. Given this definition of bladder health, it is essential to have an understanding of social processes and environmental contextual factors that may influence toilet access as a potential protective factor for bladder health.

Existing research on factors that reduce toilet access and use has largely focused on the environmental context of low-and middle-income countries or narrow age groups [3,5]. Research conducted in higher-income countries has shown that children’s concerns related to cleanliness, odor, privacy and safety may deter bathroom use [6,7,8,9]. Similar concerns about cleanliness have been shown to influence women’s use of public toilets in the United States (U.S.) [10]. In the workplace, “waiting too long to urinate” has been associated with LUTS in women [11,12], and women in professions with limited or restricted toilet access have a higher prevalence of LUTS compared to occupations with less restricted access [11,13]. Although these studies provide important insights about the availability of toilets and the factors impacting toilet access within U.S. populations, they neither apply a life course perspective nor examine experiences across diverse settings.

The purpose of this qualitative study was to explore U.S. adolescent and adult cisgender women’s lived experiences navigating toilet access and use across a variety of settings outside of the home, namely schools, workplaces, and public spaces. Unlike quantitative research which typically is powered to identify statistical significance by examining the views of a broad number of individuals from representative populations, this qualitative study is designed to explore in-depth accounts of smaller numbers of women to identify potential hypotheses about the factors influencing perceptions, beliefs, and behaviors around women’s toilet access in the U.S. This study also examines experiences of adolescent and adult women at different stages of the life course, from adolescence to older adulthood, which facilitates development of hypotheses about similar and distinct influences across different age groups and life stages. Ultimately, the findings can inform and enhance our knowledge of how to promote healthy bladder habits and function in adolescent and adult women and inform future bladder health research.

## 2. Materials and Methods

### 2.1. Overview

The Study of Habits, Attitudes, Realities, and Experiences (SHARE) is a qualitative study of the Prevention of Lower Urinary Tract Symptoms (PLUS) Research Consortium, a transdisciplinary network of seven geographically diverse research centers and a Scientific and Data Coordinating Center. PLUS was established to expand research related to the prevention of lower urinary tract symptoms (LUTS) and the promotion of bladder health in adolescents and adult women [14]. The SHARE study used focus group methodology to explore adolescent and adult women’s experiences, perceptions, beliefs, knowledge, and behaviors related to bladder health and function across the life course. This qualitative methodology was identified as best for meeting the study aim to gain insight through discourse and discussion within a small group of people who share a social context [15,16,17,18]. The full protocol has been published elsewhere [19]. This paper reports an analysis of adolescent and adult cisgender women’s lived experiences accessing and using toilets in schools, workplaces, and public spaces.

### 2.2. Participants

Participants were adolescents and adult women across a broad age range recruited at each of the seven research centers, using a variety of recruitment methods including flyers, on-line postings, word of mouth, email announcements, and referral by community partners. Applying a life course perspective, a purposeful sample of participants was recruited into focus groups stratified by age group (early adolescents: 11–14 years; adolescents: 15–17 years; young adult women: 18–25 years; adult women: 26–44 years; middle-aged women: 45–64 years; and older women: 65+ years) [20].

Participants were eligible if they were age 11 years or older, assigned female sex at birth and cisgender, spoke English or Spanish, were able to read and provide written informed consent (or assent and parental permission for those 11–14 years of age), and self-reported the absence of any physical or mental condition that would impede participation. Current known pregnancy was an exclusion criterion due to the known effects of pregnancy on LUTS. To ensure a wide range of bladder experiences, participants were included without regard to LUTS status, which was only assessed following focus group participation.

Our goal was to recruit a sample that was diverse with respect to race, ethnicity, education, socioeconomic states, physical/health conditions, LUTS status, geography (urban/rural), and language, including focus groups conducted in Spanish. Recruitment was aligned with community engagement activities of PLUS and coordinated across centers to strategically determine the composition of each focus group and ensure overall study diversity.

The study was approved by the University of Pennsylvania Institutional Review Board (IRB), which served as the central review board for six of the seven centers, and a local university IRB at the remaining site. All participants signed informed consent forms and received $50 gift cards for their participation in the focus group.

### 2.3. Procedures

Between July 2017 and April 2018, focus groups were conducted within specified age groups to encourage comfortable and open communication among participants. The sessions lasted about 90 min and were conducted in community settings, such as libraries, universities, community organization meeting spaces, and churches. Focus groups were conducted by female moderators who received standardized training in the qualitative research principles adopted by the PLUS Consortium, best practices for focus group research, and the SHARE study protocol (see Hebert-Beirne, et al., for full description of moderator characteristics) [21]. Participants did not have prior relationships with the research staff, however at three research centers, participants were recruited from community partners of the research center.

Each session followed a semi-structured focus group guide informed by the PLUS conceptual framework, which was based on the social ecological model and incorporated a life course perspective [2]. The guide was arranged in five sections, consisting of 18 core items (with probes) covering the following domains: healthy bladder beliefs/attitudes, bladder knowledge acquisition, LUTS experience and care seeking, terminology, and public health messaging. The guide was piloted through mock focus groups coordinated with community partners.

Focus groups were audio-recorded and professionally transcribed verbatim. In addition, trained female investigators observed the sessions and noted analytic insights using a field note guide for documenting nonverbal communication and characterizing the focus group dynamics. After each focus group session, participants completed self-administered measures to characterize the sample in terms of demographics and LUTS status, as measured by the LUTS Tool in adults [22] or ICIQ CLUTS Survey in adolescents [23].

### 2.4. Data Analysis and Interpretation

Data analysis and reporting were conducted in accordance with the Consolidated Criteria for Reporting Qualitative Research (COREQ) guidelines (See Supplementary Table S1) [24]. Focus group transcripts and field notes were entered into Dedoose, a software program for facilitating the organization and coding of non-numerical data. We employed a protocol-driven, systematic multilevel approach to codebook development and data analysis. The first level of analysis consisted of a deductive directed content analysis (DCA) [25,26]. DCA is used when current theory or previous evidence (in this case the PLUS conceptual framework [2]) needs further description [26]. Consistent with DCA, we organized the large body of data into a coding schema aligned with social ecological components of the PLUS conceptual framework, which informed our interview guide. We then used a deductive approach to explore textual data for insights relevant to the research question, with the goal of validating and extending knowledge in the area of interest. The second level of data analysis expanded the coding structure to include the valence of the level one codes and include subcategories to document extensions or variations on the initial coding categories. The third level of data analysis utilized a phenomenological inductive lens to articulate emerging insights about social processes and cultural assumptions shaping the content of the focus group narratives.

A 4-member transdisciplinary team of researchers and two trained data coders carried out the first and second levels of data analysis. At the outset of the interpretative process, team members engaged in a reflexive process, describing their disciplinary perspectives and potential biases. This process supported the use of a transdisciplinary lens which engaged all analytic team members in the process of the analysis. All study investigators conducted the third level of data interpretation via an inductive process guided by team science and informed by a transdisciplinary perspective that utilized the integrative expertise and experience of both male and female social and behavioral scientists, medical clinicians and interventionists, public health researchers and educators, and community advocates. The key mechanisms of data interpretation included data immersion (with coded data and transcripts), investigator reflexivity and team dialogue, which led to the development of themes [27].

To ensure trustworthiness of the analysis, study investigators directly supervised coders and trained them to identify inconsistencies with codes and emerging themes. Study investigators also directly observed the focus groups and maintained a detailed log of coding and analytic decisions [28]. A validation process was performed wherein results were presented to the focus group moderators from all sites, community members at one site, and PLUS research investigators who were not directly involved in the study to gain further insights and validate themes.

The present analysis focuses on interpretive analysis of the inductively-derived codes of “Accessing Toilets”, “School”, “Workplace”, and “Community Spaces”. The unit of analysis was the group (rather than the individual), and we present data that reached saturation about accessing and using toilets in schools, workplaces, and public.

## 3. Results

A total of 44 focus groups were conducted with 360 participants. The number of focus groups conducted varied by age category, with more focus groups being conducted within the older age categories per the study protocol (see Table 1). The participants ranged in age from 11–93 years and represented a diverse sample across racial, socio-economic, and geographic distributions (see Supplementary Table S2). Overall, 76.4% of the participants reported LUTS in the past week, with younger participants having the lowest prevalence (44.4% in 11–14-year olds) and oldest participants having the highest prevalence (87.6% in 65+) of one or more symptoms.

Through the process of interpretive analysis, several themes related to toilet access and use emerged. Gatekeepers (defined as individuals who control access to toilets) and self- restricting toilet use (deciding not to use the toilet despite biologic need to urinate, often in response to the internalization of external norms) played a prominent role in toilet access and use across age groups. An emergent theme that also arose was gender disparities in toilet access in public spaces.

### 3.1. Gatekeepers

#### 3.1.1. Schools

Gatekeepers were described by both adolescent and adult women across the life course as being particularly prominent in limiting toilet access in school settings. Adolescents identified teachers as the primary gatekeepers in the school setting; however, security guards and hall monitors also performed this role:

“Our engineering teacher, he only, he lets you go for only five minutes, and if you take one minute more, then he’s not gonna let you go to the bathroom for another week.”

*—11–14 age group*

“I don’t want like to go to the bathroom with a security guard having to unlock the door, especially if I go frequently, they start to ask questions too, when it’s not their business.”

*—15–17 age group*

Participants from other age groups provided retrospective accounts of how gatekeepers had limited their access to toilets in elementary or secondary school settings:

“If you raise your hand and, and say, “Can I go to the washroom?” Sometimes the teacher would say ‘noooo’, …maybe that kid had a problem and he really needed to go, so he had to sit there until it was time to go.”

*—65+ age group*

“I had a similar experience when I was a kid, so 35, however, many years ago. …it was extremely embarrassing because I went because the, the teacher wouldn’t let me get up and go and so there I was in third grade with a puddle under my chair and then that creates its own stigma for the rest of my time at that school.”

*—26–44 age group*

Participants offered multiple explanations for gatekeeping in schools, rationalizing the process as a means of supporting optimal student learning. For example, adolescents described how gatekeepers wanted to limit time out of the classroom to encourage a “focus on [school] work”.

“I know in my Spanish class, we is not allowed to go to the bathroom at all because she always say that we have to focus on work [rather] than leaving out the class.”

*—11–14 age group*

“They told me I can’t [use the bathroom] because I’ll miss something, so I just stay.”

*—15–17 age group*

They also shared perceptions that gatekeepers assumed students would engage in misbehavior during a bathroom break:

“They think maybe you’re like playing outside, so sometimes they don’t let you go to the bathroom.”

*—11–14 age group*

Although gatekeepers limiting toilet access to control the potential for misbehavior could be viewed as unfair, some adolescents acknowledged that students may indeed engage in behaviors other than toileting in the restroom:

“Well, first of all, since like most of the girls go to the bathroom just to chat, they made that rule that only we can go once a day.”

*—11–14 age group*

“Yeah. A lot of people don’t use the bathroom. They just stay on their phones.”

*—15–17 age group*

“School–wise, a lot of people skip classes in the bathroom and a lot of people are in the bathroom to not go to the bathroom. So think that when schools do that [limit toilet access], they’re trying to prevent that.”

*—15–17 age group*

These sentiments were shared by adult women who recalled teachers with similar assumptions about misbehavior in the bathroom.

“The teacher made me sit there and wait, like, I guess they thought maybe you were playing around, you just wanted to get out of class, but no, some of us really had to get up and go to the bathroom. You tell us to sit there and hold it.”

*—45–64 age group*

#### 3.1.2. Workplaces

While the role of gatekeepers was a salient theme across the life course in reference to toilet access in schools, adult women varied with respect to whether they perceived that gatekeepers limited access to toilets in occupational settings. Some participants described how managers or supervisors served as gatekeepers for employee toilet access:

“Sometimes managers micromanage it. They figure you’re getting up every 15–20 min to go to the bathroom, and they will tell you that you only have two 15–minute breaks a day.”

*—45–64 age group*

“You have to badge in and badge out of the bathroom because they’re watching how many breaks you take.”

*—45–64 age group*

Compared to the discussions that arose around the school setting, overall there was less discussion about why gatekeepers in workplaces limit access to toilets. Instead, some women described how toilet access was limited to scheduled breaks or lunch.

“Where I work, you can only pee on your breaks.”

*—18–25 age group*

“You had certain breaks that you could go and that’s when you could go.”

*—45–64 age group*

“You have to hold it until there’s a scheduled break, so you don’t have the opportunity to go when you need to.”

*—65+ age group*

Although some participants experienced instances wherein managers controlled access to toilets, some participants indicated they had agency in accessing and using toilets in their workplaces:

“I have no limitations on time or pressure of using the restroom in my job.”

*—18–25 age group*

“My work does not schedule your restroom breaks, you can go as needed, which is good.”

*—25–45 age group*

#### 3.1.3. Public Spaces

Women of all ages, including adolescents, described public toilets located in commercial and non-commercial spaces. The presence of gatekeepers was not described in non-commercial toileting spaces, which included “porta-potties” and public restrooms in parks or rest stops. However, participants described a range of experiences in which store employees served as gatekeepers in commercial spaces, including small and large retail establishments (e.g., corner stores, “big-box” stores, and grocery stores). Participants explained how access to the toilets in retail establishments was dependent upon one’s ability to purchase goods, and in these cases, the store employees served as the gatekeeper:

“…what is sad is that it’s women who are at the store or business, and you tell them, ‘can I please use your bathroom? It’s an emergency?’ ‘No, you have to be a client; will you be buying something?’ ‘No, I need to use the bathroom.’ ‘No, you can’t.’ ‘It’s an emergency.’ ‘No.’”

*—45–64 age group*

Businesses’ concerns about liability was also described as the basis for gatekeepers’ restricting access to toilets in commercial spaces:

“…two weeks ago, I was in a discount grocery… they said, ‘Oh, no. You can’t use the bathroom. We just cleaned it.’ And she [i.e., the woman who wished to use the toilet] was like desperate. This woman was like, ‘Please, please. I’ve got to go. I don’t know where to.’ And they said, ‘No, if you would slip and fall on the wet floor, you could sue us,’ and they would not let her use the bathroom. She was begging to use the bathroom.”

*—65+ age group*

In some cases, the employee would allow access to the toilet:

“It’s like the home store where you go in and there’s just a bunch of random stuff. So, I was like listen, I really need to use the bathroom. Do you guys have a bathroom? And I’m pretty sure it’s not a public bathroom but he saw that I really needed to pee, so he’s like yeah, you can use the bathroom in the back and then there’s just a bunch of like boxes.”

*—15–17 age group*

Participants also noted positive responses to certain retail establishments whose employees allowed access to toilets, and they generally had favorable attitudes towards these locations:

[In reference to large retail stores] *“It’s like if you have to pee, either like go inside … and they’ll let you use the bathroom because they don’t care.”*

*—25–64 age group*

[In reference to a national chain restaurant] *“It was almost like a community center, so… their bathrooms were open to everybody, which was good.”*

*—65+ age group*

In summary, the process of toileting was challenged by gatekeepers in schools, workplaces, and commercial spaces. Teachers, managers and store employees served as gatekeepers. In commercial spaces, participants described a range of experiences where staff in those settings were either permissive or restrictive in allowing access. In each setting, gatekeepers’ actions could result in adolescent and adult women suppressing their biological need (i.e., holding their urine).

### 3.2. Self–Restricting Toilet Use

#### 3.2.1. Schools

Some adolescents in secondary school and young adult women in college made decisions not to go to the bathroom to maximize learning opportunities. Presumably, external expectations to focus on schoolwork were internalized resulting in decisions to not use the toilet:

“Like at school, when I don’t want to miss a certain like thing they’re going to tell us, I just kind of wait a little bit.”

*—11–14 age group*

“I really don’t want to miss the lesson because it’s more that I don’t know, and I don’t want to get behind. So, if I have to use the bathroom, then I’ll just wait.”

*—15–17 age group*

“Like, in college, I haven’t had a problem with being able to go to the bathroom but it’s just like I don’t want to miss information, in my like hour and a half long class, so I’ll sit there and then like rush to the bathroom as soon as the lecture’s over and, like, normally I should have gone but I didn’t wanna miss the information that I will probably be tested on.”

*—18–25 age group*

In these cases, instead of being limited by gatekeepers, adolescents and young adult women restricted their own behavior when they prioritized school work over responding to bladder cues.

#### 3.2.2. Workplace

The notion of self-restricting use was prominent in the workplace in relation to a variety of professions and among women in a variety of age groups. This behavior was demonstrated as expectations to fulfill job responsibilities were internalized, resulting in decisions to not use the toilet:

“I worked at like a dining hall type situation and since it was me and maybe one other person, we couldn’t just leave whenever we wanted to.”

*—18–25 age group*

“I was working with kids, preschool teacher. So, when you got to go, you can’t run out and leave the kids.”

*—45–64 age group*

“Well, you’re so busy with your patients and stuff, you don’t take the time to go.”

*—65+ age group*

Self-restricting toilet use was even expressed by this adolescent participant when describing how she prioritized her summer job responsibilities over her need to toilet:

“When I work in the summer, I have to work with kids and you cannot leave the kids unattended, so you can’t just always put yourself first… working somewhere where you have to be on your feet or like interacting with people, you can’t just leave your station when you have to, like, you kind of have to put your needs and necessities on the back burner when you’re in the work setting.”

*—15–17 age group*

In these instances, women did not describe an external pressure from a gatekeeper to prioritize their job responsibilities; rather, foregoing a visit to the restroom and holding urine seemed to be an act of self-restricting use based on internalized workplace social norms and rules.

#### 3.2.3. Public Spaces

Self-restricting use in public spaces was in response to perceived lack of cleanliness of public toilets, especially those not located in commercial spaces. In contrast to women’s discussion of the availability of clean and acceptable toilets in some retail establishments, porta potties and to some degree, public restrooms in parks or rest stops, were almost always portrayed as aversive, due to their lack of cleanliness, unpleasant smell, and perceived harboring of germs. These notions were manifested in language such as “*they’re so disgusting*”, “*gross*”, “*it’s just dreadful*”, “*they’re dirty, they stink*” and “*I feel like it’s a, like a small germ place, germ, germ filled place*”. Participants restricted their own use of toilets in response to lack of cleanliness:

“Like a dirty gas station bathroom then I can’t go.”

*—18–25 age group*

“I enter and that thing is in such condition, almost makes one cry, I prefer to hold it in and I turn around.”

*—65+ age group*

“Sometimes when you be out shopping or something and then you get the urge to go to the bathroom, you’ll find where there’s a bathroom and it’s not clean. You rather not, that’s right, you’d rather not go.”

*—65+ age group*

Another manner by which self-restricting use was manifested was through the internalized norm of needing to purchase goods when accessing public toilets. For example, one woman shared that she purchased goods when using public toilets in retail establishments, even when not explicitly asked to buy anything to gain access to the toilet:

“My husband and I travel a lot and we always look for fast food places because you can always go in there and use the bathroom. We usually buy a thing of iced tea because we feel bad about using the bathroom and not buying anything.”

*—65+ age group*

### 3.3. Gender Disparities in Public Toilet Access

Across their life course, participants observed differences in men’s and women’s toilet access in public spaces. They frequently recounted longer lines for women’s than men’s restrooms, believing that urinals allowed men to access toilets more efficiently.

“Even at the gas station; even at, like when you’re on a trip or something, you started to get the Stop and Go or whatever, it’s like a long line at the ladies’ room and the men’s room is like nobody, you know.”

*—45–64 age group*

Some adult women managed their voiding needs by using the men’s restroom as an alternative to the women’s restroom lines:

“I know I used the men’s restroom multiple times just from the women’s line just being too long and you’re like yeah, I’m not going to wait, because there’s no one even in the men’s room, so like I know I’ve done that a lot of times, but I’ve never heard of a guy using the women’s restroom.”

*—18–25 age group*

“I go to the men’s room no shame. I just scoot right in to the men’s room because the men’s room never has a line.”

*—45–64 age group*

Participants discussed a variety of reasons why women’s public restrooms have longer lines than men’s’ restrooms. For example, menstruation might lead to the need for females to spend a longer time in the restroom than males:

“It does make you think, though, like why do like, of course, women have menstruation and stuff so like they might have to take care of that, but why is there longer lines for women than males?”

*—18–25 age group*

Adolescents and young adult women explained how it was challenging to undress while toileting, contributing to a longer time spent in the restroom. This was relevant to clothing items such as bathing suits, jumpers, dance leotards, and uniforms:

“I’m also in marching band and sometimes at football games, it’s a pain to get out of our entire uniform.”

*—15–17 age group*

This contrasted with perceptions of men’s lack of clothing impediments, which facilitated easy toileting:

“They don’t even have to get undressed. They, they don’t. They just whip it out.”

*—18–25 age group*

While younger women noted challenges related to clothing, among older adult women longer lines for women’s toilets were attributed to LUTS, such as the need to use toilets more frequently or for a longer period of time:

“You know I always think when I was young, I said why is this women’s restroom so long? The men don’t have nobody in line but us women, we’re always lined up at a, if it’s an arena, arena or anything like that. And I did not realize that’s what it was until I got older that a lot of us women have problems with our urine, the bladder.”

*—65+ age group*

Adolescent and adult women expressed the notion that although it was acceptable for males to urinate outside or in public, females were socialized at a very early age to urinate in private. When traveling, parents of young daughters were more restrictive than parents of young sons when it came finding toileting locations for their children.

“Us as women, we cannot go just in any place. When you have a boy, you can have him pee, but not a girl.”

*—45–64 age group*

This early socialization led to social norms that persisted later in the life course. For example, when traveling, some women chose to wait to urinate in public restrooms rather than “the side of the road” or “in the woods”.

“We can’t just pull over and pee, like we’re not dudes. We got to wait until the next rest stop, which could be miles away.”

*— 15–17 age group*

While participants described their observations of gender disparities in socialization about norms for privacy when using the toilet or voiding compared to males for a variety of reasons, several also described the socialization process as being unfair.

## 4. Discussion

This qualitative study explored U.S. adolescent and adult women’s experiences accessing toilets in schools, workplaces, and public spaces. Gatekeepers played a prominent role in controlling toilet access in school and in some occupational and public settings. Self-restricting toilet use based on internalized norms led women to balance toileting with school and job responsibilities. In public spaces, self-restricting toilet use was in response to lack of cleanliness or a perceived need to reciprocate toilet access with purchasing goods. Similar to previous studies conducted in low- and middle-income countries [29,30,31], we also found that, across the life course, adolescent and adult cisgender women perceived gender disparities in the ability to easily access public toilets. By using qualitative research to examine experiences across the life course, from adolescence to older adulthood, our findings point to future bladder health research needs regarding women’s toilet access in the U.S.

A striking finding in the data was the prominence of the gatekeeper in regulating adolescents’ toilet access in school settings. In contrast, gatekeepers had a more variable influence on regulating women’s toilet access in workplace settings. A few studies have described teachers serving as gatekeepers among children with urinary incontinence [32,33]; however none have described both adolescents and adult women who had attended school up to 40 years ago recalling similar experiences of having gatekeepers limit their access to toilets. This finding suggests that limited toilet access during childhood and adolescence may be a universal experience among generations of women in the U.S. Previous research has shown that young people adapt to an unpleasant school toilet environment or limited access by delaying urination and limiting fluid intake, both of which have been shown to contribute to incontinence, dysfunctional elimination and increased risk for urinary tract infection [3,7,34]. Our data suggest that most women in the U.S. may have had toilet access restrictions during childhood and adolescence, with many additionally self-restricting use based on internalized norms. Quantitative research is needed to determine the prevalence of toilet access restriction during developmental life stages of childhood and adolescence and both its immediate and long-term impact on bladder health across the life course. These data can inform future research that focuses on the development of survey instruments to identify the prevalence and correlates of gatekeeping practices within the U. S. and to examine cultural variations in the process of gatekeeping among international populations. Research also is needed to enhance our understanding of the process of gatekeeping in schools, workplaces, and other public spaces. Research has shown that U.S. teachers receive little training on how to manage voiding in school children [35,36,37], however a Belgian study has demonstrated that staff education about toileting is associated with improved student hydration status and increased acceptability of school toilets [38]. Thus, future public health studies are needed to determine how to best educate U.S. school gatekeepers, teachers in particular, about the important role of toilet access in maintaining bladder health.

Our study also describes the phenomenon of self-restricting toilet use in U.S. schools or workplaces, which was demonstrated by limiting one’s own use of toilets in response to internalization of external norms regarding expectations to be present for education and work tasks. A study of nurses in Australia similarly showed that nurses prioritized a work culture of “patient-first” over their own self-care and voiding [39]; however, our data suggest that this behavior may occur across a variety of professions as well as among students. Future research is needed to understand why U.S. women may self-restrict toilet use and prioritize school and workplace responsibilities over the biologic need to urinate.

In public spaces, self-restricting use largely arose from perceived lack of cleanliness. Previous research has suggested that women and men have unique expectations when accessing public toilets, and adolescent and adult women highly value cleanliness [10,30,40,41]. Although, to our knowledge, little published research explores cisgender men’s acceptance of porta-potties and public toilets, our data suggest that many public toilets are not acceptable to cisgender women, and their current availability may not improve toilet access and use in this population. Our data also suggests that the perception of lines for women’s toilets may serve as a barrier to accessing women’s restrooms. Some women described using men’s toilets to avoid the lines, illustrating how biologic needs may override social norms regarding gender-specific bathrooms. Since U.S. adolescent and adult women perceive differences in men’s and women’s ability to freely access public toilets, future quantitative research is needed to determine the extent of these potential disparities in nationally representative samples of women and men in the U.S. that are geographically and demographically diverse. Of note, this study focuses on the experiences of cig-gender women, and research with men or gender non-conforming or transgender individuals is also needed to understand the entire scope of issues related to toilet access disparities. The World Health Organization Sanitation guidelines view accessible sanitation as a human right, further highlighting the need to understand the extent of potential toilet access disparities in the U.S. [1].

Finally, our findings provide novel insights about the role of businesses in promoting toilet access in public spaces. Gatekeeper influences on toilet access were reflected by women’s descriptions of employees requiring store purchases to access toilets. Self-restricting use was reflected by internalizing norms to purchase goods in an effort to access toilets. While it is not within the scope of this study to examine ethical or legal issues regarding businesses’ toileting access practices in the U. S., we believe these data highlight the need for future research in this area. Future research and policy efforts may need to include the business community in designing accessible public toilets or in reconsidering rules about bathroom access.

This study has several limitations. By its nature, qualitative data are not designed to inform inferences about causality. Although this study included a geographically and demographically diverse sample of female individuals in the U.S., expressed perspectives may not represent the views of all subgroups of women, and future research is needed to better understand racial/ethnic differences in these perceptions and whether unique factors exist for certain ethnic groups which were not highly represented in the sample (e.g., American Indian/Native American, Asian, Native Hawaiian, and other Pacific Island women). Further, the specific barriers to toilet access identified in our study may or may not be applicable to women living in countries other than the U.S. Finally, our findings regarding toilet access are based on adolescent and adult cisgender women’s voices only. Research with men or gender non-conforming or transgender individuals may yield different, but equally compelling accounts of gatekeepers and self-restricting toilet use.

## 5. Conclusions

Our findings illustrate that U.S. adolescent and adult women encounter gatekeepers and self-restrict toilet use in schools, workplaces and public places. These experiences potentially impact bladder health. Gatekeeping in school settings is similar across age cohorts, suggesting the deeply rooted and institutionalized nature of this practice. Similarly, gatekeeping in the workplace is attributed to commonly shared assumptions about the importance of prioritizing productivity over toileting needs. The notion of self-restricting toilet use speaks to the nuanced nature of toileting access and points to the complex ways that individuals interact with the toileting environment in schools, workplaces, and public spaces. These findings can inform future bladder health research aimed at understanding and promoting healthy bladder habits and function in U.S. adolescent and adult women across the life course.

## Figures and Tables

**Table 1:** Numbers of Participants and Focus Groups, Total and by Age Category.

Number (n)	Total	Age Category					
		11–14	15–17	18–25	26–44	45–64	65+
Number of participants, total and by age category	360	18	26	51	72	104	89
Number of focus groups, total and by age category	44	4	4	6	9	11	10

## Supplementary Materials

The following are available online at https://www.mdpi.com/1660-4601/16/18/3338/s1, Table S1: COREQ (COnsolidated criteria for REporting Qualitative research) Checklist, Table S2: Participant Characteristics.
